# Pilgrims satisfaction with ambulatory health services in Makkah, 2008

**DOI:** 10.4103/1319-1683.74331

**Published:** 2010

**Authors:** Ibrahim A. Al-Hoqail, Abdelshakour M. Abdalla, Abdalla A. Saeed, Nasir A. Al-Hamdan, Ahmed A. Bahnassy

**Affiliations:** *Department of Dermatology, College of Medicine, King Saud University, Riyadh, Kingdom of Saudi Arabia*; 1 *Department Community Medicine, King Saud Bin Abdul-Aziz University for Health Sciences, King Fahad Medical City, Riyadh, Kingdom of Saudi Arabia*

**Keywords:** Ambulatory health services, Hajj pilgrims, Makkah

## Abstract

**Objective:**

The main objective of this study was to assess the level and correlates of patients’ satisfaction with ambulatory health services provided for pilgrims during Hajj period in 2008.

**Materials and Methods:**

This was a facility-based, cross-sectional study conducted in the Makkah region during the Hajj season in December 2008. A two-stage technique was used to select 500 patients from those who attended the ambulatory health services. One hundred subjects were selected by systematic random sampling (every fifth) from each of the five hospitals included in the study and asked to fill in a pilot-tested self-administered questionnaire. A total of 487 questionnaires were analyzed. Descriptive statistics and t-test, Mann Whitney test and ANOVA, or Kruskal-Wallis test was used as appropriate after checking for normality. Level of significance level was set to be <0.05 throughout the study.

**Results:**

From 478 subjects analyzed, 390 (81.6%) were man, 345 (72.2%) were married, 28.9% had either intermediate or high secondary school education, and 2.4% were skilled laborers. The total satisfaction score for health facilities was 20.45 ± 4.03 of 25. The satisfaction scores were 20.15 ± 4.7 of 25 for patient satisfaction with physicians and 21.35 ± 4.5 for patient satisfaction with paramedical personnel. The overall satisfaction score was 61.5 ± 4.5 of 75 points. There were significant relations between total satisfaction of health facilities with education level and with occupation (*P* = 0.012, 0.001, respectively). The total satisfaction of patients with physicians was significant only with education level. The overall satisfaction score had a significant relation with occupation (*P* = 0.03), but a borderline relation with the education level (*P* = 0.056).

**Conclusion:**

Satisfaction with ambulatory Hajj health services is acceptable. Some physicians and waiting area services need special attention to improve satisfaction levels with ambulatory health in the subsequent Hajj seasons.

## INTRODUCTION

Measuring satisfaction in many communities has increased with the development and growth of the consumerism. Client satisfaction surveys have been used extensively in developed countries and have grown substantially in many developing countries.[1–5] The last two decades have witnessed an increase in the attention given to users’ satisfaction with health services.[5] This reflects the value of consumers’ opinions about the quality of a product or service. Patient satisfaction is an important element in evaluating the quality of health care services, and in predicting patients’ behavioral patterns after receiving the services. In addition to measuring client satisfaction, such studies can identify facility attributes or practices that increase satisfaction and utilization, which may lead to favorable outcomes.

Several tools were used to study consumer satisfaction. These included interviews and open-ended questions which produced detailed information that is usually difficult to analyze. More structured multiple-item questionnaires with Likert scale response categories produced data which can be handled easily but require close attention to validity and reliability.[6–12]

Hajj (Pilgrimage) is a special season where more than two million Muslims from more than 150 countries gather every year at the holy shrine (Al-Mashaer) to perform this important ritual of Islam. Hajj is performed in Makkah and Al-Mashaer in the Kingdom of Saudi Arabia (KSA), which includes Mina, Mozdalifah and Arafat. The ritual starts on the 8th and ends on the 13th day of Dhul Hijjah, the 12th month of the lunar Islamic calendar year. It corresponded to December 5 to 11, 2008 in this study. The government of the KSA through Ministry of Health provides free health services for all pilgrims during Hajj period.

The main objectives of this study were to assess the level and correlates of satisfaction of pilgrims attending Hajj ambulatory health services. To the best of our knowledge, this was the first survey of its kind in the Kingdom to focus on ambulatory Hajj health services. We hope that the findings of this study may be of help in improving the ambulatory health services during Hajj season.

## MATERIALS AND METHODS

This was a facility-based, cross-sectional study conducted during the Hajj season for one week from December 5 to 11, 2008. A two-stage sampling technique was used. In the first stage, five hospitals (of the ten hospitals) providing health services for pilgrims in Makkah and Mina were selected randomly. In the second stage, a systematic random sampling with five-interval spacing was used to select 100 patients from each hospital, giving a total of 500 patients of those who attended the ambulatory health services during the time of the study. Selected subjects completed a predesigned, pilot-tested self-administered questionnaires.

The questionnaire consisted of three main parts beside the demographic variables. The first part consisted of questions related to satisfaction with the conformability of the patient in the health facility; the second part contained questions measuring satisfaction with the physician; and the third part measured satisfaction with other health team personnel. Each section had five subcategories. All questions measuring satisfaction were rated on a Likert scale of five points ranging from 1 (lowest satisfaction) to 5 (highest satisfaction scores), so the maximum satisfaction score for each section was 25 points and maximum overall satisfaction score for the three sections was 75 points.

The questionnaire was reviewed by three specialists in community medicine, nursing, and health service administration to assess face validity, and resulted in an agreement on 14 of the 15 items. Reliability of the questionnaire was assessed using Cronbach’ alpha coefficient. It was 87.0%. The data were collected by trained Medical students from faculty of Medicine, King Fahad Medical City, who were available to help illiterate and non-Arabic speaking pilgrims in completing the questionnaires. Verbal and written confidentiality was assured and patients were informed that the data would be used only for the stated research purposes, and that their participation was totally voluntary.

Permission for the study was obtained from authorities concerned. All data were checked for completeness, entered into a personal computer, and analyzed using SPSS version 17. Descriptive statistics and t-test, Mann Whitney test and ANOVA, or Kruskal-Wallis test was used as appropriate after checking for normality. Significance level was set as <0.05 throughout the study.

### Definition

Hajj is one of the five pillars of Islam. It is a set of acts of worship to be performed in and around Makkah at least once in a lifetime by every Muslim who satisfies certain conditions.

## RESULTS

Of the 500 subjects who participated in the study, 22 (4.4%) were excluded because their responses were missing. Of the remaining 478 subjects, 390 (81.6%) were man, 345 (72.2%) were married, 133 (28.9%) had either intermediate or high secondary school education, and 127 (27.4%) were skilled laborers [Table 1].

**Table 1:** Demographic characteristics for the studied Sample*

Variable	Number	%
Sex		
Male	390	81.6
Female	88	18.4
Marital status	345	72.2
Married		
Single	115	24.1
Widow	9	1.9
Divorced	6	1.3
Education		
Do not read and write	81	17.6
Primary school	86	18.7
Intermediate or secondary	133	28.9
Graduate	111	24.1
Postgraduate	49	10.7
Occupation		
Unemployed	71	15.5
Student	54	11.7
House wife	43	9.3
Unskilled labor	63	13.6
Skilled labor	127	27.4
Professional	104	22.5

* Totals not equal the total sample 478 because of some missing values

Table 2 shows the descriptive summary of the studied items. Total satisfaction score for health facilities was 20.45 ± 4.03, while it was 20.15 ±4.7 for satisfaction with the physician, and 21.35 ± 4.5 with the paramedical personnel, all of 25 points. The overall satisfaction score was 61.5 ± 4.5 of 75 points [Table 2].

**Table 2:** Mean satisfaction score for the studied items

Variable	Mean ± Standard deviation
The health facility	
Seats	4.15 ± 0.893
Waiting area	4.04 ± 0.988
Ventilation	4.16 ± 0.948
Cooperation with patient	4.13 ± 1.064
Reception	4.13 ± 1.077
Total satisfaction score for the health facility	20.45 ± 4.026
The physicians	
Listening to the patients	4.37 ± 0.842
Knowing	4.30 ± 0.893
Clinical examination	3.86 ± 1.193
Explain the diagnosis to the patient	3.92 ± 1.171
Reply	3.94 ± 1.162
Total satisfaction score for the physicians	20.15 ± 4.680
Medical allied services	
Pharmacy site	4.32 ± 0.958
Medication availability	4.31 ± 0.986
Pharmacists explaining the doses	4.32 ± 0.914
Pharmacists dealing with the patient	4.32 ± 0.947
Nurses dealing with the patient	4.41 ± 0.846
Total satisfaction score for the other health	21.35 ± 4.497
team members	
Mean total satisfaction score	4.09 ± 0.865
Overall satisfaction score for all studied	61.47 ± 11.400
items	

Table 3 shows the relation between total satisfaction scores for each part with demographic variables. Significant relationship was noticed between total satisfaction with the health facilities and each of educational level and occupation (*P* = 0.012, 0.001, respectively), whereas no significant relationships were noticed with marital status and gender. With respect to the total satisfaction of the patients with physician, it was significant only with educational level (*P* = 0.042). With regard to satisfaction score of the patients with the other health team members, there was no statistical significance with any of the demographic variables. For the overall satisfaction score, a significant difference was noticed with occupation (*P* = 0.03), whereas there was borderline significance as regards to the educational level (*P* = 0.056).

**Table 3:** Relation between satisfaction scores and some demographic variables

Item	Score 1 mean ± SD	Score 2 mean ± SD	Score 3 mean ± SD	Total score mean ± SD
Education	*P* = 0.012	*P* = 0.042	*P* = 0.32	*P* = 0.056
Do not read and write	21.2 ± 4.0	21.34 ± 4.01	21.6 ± 4.8	63.3 ± 10.9
Primary school	21 ± 3.8	20.1 ± 4.7	21.9 ± 5.1	62.65 ±11.4
Intermediate and secondary	20.4 ± 4.1	19.4 ± 5.1	20.7 ± 4.6	60.1 ± 12.3
Graduate	20.5 ± 4.1	20.5 ± 4.4	21.7 ± 4.1	62.92 ±10.3
Postgraduate	19.1 ± 3.7	19.8 ± 4.81	21.3 ± 3.7	59.1 ± 11.5
Occupation	*P* = 0.001	*P* = 0.27	*P* = 0.04	*P* = 0.028
Unemployed	21.1 ± 3.7	20.8 ± 4.4	21.3 ± 4.8	62.6 ± 11.5
Student	19.9 ± 4.3	20.8 ± 4.9	21.4 ± 4.5	61.7 ± 12.7
Housewife	20.6 ± 4.3	19.8 ± 4.8	21.3 ± 5.3	61.2 ± 12.7
Unskilled labor	22.1 ± 2.9	20.9 ± 3.9	22.8 ± 3.6	65.5 ± 8.3
Skilled labor	20.4 ± 4.3	19.8 ± 4.9	21.5 ± 4.3	60.83 ±11.8
Professional	19.6 ± 4.1	19.6 ± 4.8	20.5 ± 4.6	59.6 ± 10.9
Marital status	*P* = 0.42	*P* = 0.5	*P* = 0.52	*P* = 0.62
Married	20.6 ± 4.0	20.12 ± 4.7	21.5 ± 5	61.6 ± 11.5
Single	20.03 ± 4.1	20.2 ± 4.8	21.1 ± 4.4	61.2 ± 11.3
Widow	21.2 ± 4.3	22.1 ± 2.8	20.9 ± 4.3	64.2 ± 4.5
Divorce	19 ± 3.2	10.5 ± 1.8	19 ± 4.4	56.5 ± 8.9
Sex	*P* = 0.27	*P* = 0.35	*P* = 0.6	*P* = 0.49
Male	20.54 ± 3.9	20.25 ± 4.6	21.4 ± 4.4	60.7 ± 11.2
Female	20.02 ± 4.2	19.7 ± 4.9	21.1 ± 4.7	60.6 ± 12.2

## DISCUSSION

Consumers’ satisfaction with health services provided has been widely studied mostly in developed countries. Patients’ perceptions are valued and are used to measure overall quality and an outcome of consultations and other encounters between providers and consumers. Satisfaction with ambulatory care has significantly influenced consumers’ behavior in looking for health care, compliance with treatment, and return to the same care facility.[13,14] Health care providers must consider whether patient expectations of their services can be managed and if so, how this can be done. Dissatisfaction with health care services could be reduced if consumers knew what to expect, and their views and suggestions on what is actually provided are taken seriously.

The overall mean consumers’ satisfaction with ambulatory health services provided in this study was 61.5 of 75 points (82%). Hajj is a ritual performed only in Saudi Arabia and the authors are not aware of any published studies on satisfaction with ambulatory Hajj health services. This discussion will focus mainly on comparing our results with studies in ambulatory health settings in the Kingdom and other countries.

Previous studies in the Saudi Arabia, some neighboring countries, and other Muslim countries reported overall satisfaction scores ranging from 60 to 90% with Primary Health Care settings.[15–22] The relatively high satisfaction in Hajj may be due to the different nationalities of the consumers. The service is free and some consumers from poor developing countries with relatively poorly developed health services are likely to respond much more favorably. Also, Hajj is a religious activity during which those performing it mostly have peace of mind, are accommodating, and generally less critical and less demanding. However, user surveys, particularly exit surveys, typically uniformly show high satisfaction with services. One likely reason is ‘courtesy bias,’ whereby respondents are reluctant to express negative opinions resulting in an overestimation of satisfaction.[23]

Consumers with lower educational level and those working as unskilled laborers were significantly more satisfied than those of higher educational level working in skilled jobs. All other variables studied were not significantly related to the overall and differential satisfaction with services. It appears that the less-educated and unskilled laborers were less demanding and their expectations were much lower than those of the highly educated, skilled, and professional persons.

Satisfaction with physicians’ services in this study scored the lowest satisfaction level compared with allied health and reception services. However, this satisfaction score is better than satisfaction scores with physicians in ambulatory settings in Saudi Arabia (2.31–2.56 points of 5 (46.2–51.2%))[17] and Kuwait City in Kuwait (2.21 points of 5 [44.2%]).[20] Satisfaction with physicians’ services significantly affects satisfaction with health services, particularly ambulatory services. Areas of physician service which need attention include clinical examination, giving explanations, and responding to queries. Studies have confirmed that physicians can enhance satisfaction by spending a little more time to talk to the patient to exchange views and give better explanation and information.[24–26] Training in communication skills increases open discussion and improves physician’s sensitivity to patients and their general satisfaction.[27] Clinical examination can be improved by increasing the number of physicians at the clinics, so that more time can be spent by the doctor to examine each patient.

Marital status, age, or gender was not significantly associated with all services. Other studies have reported that older patients tended to have significantly higher satisfaction with services provided.[19,28,29] Older subjects were generally more conservative and less demanding than younger ones. Gender and marital status were not significantly associated with satisfaction in this study, which is in accord with findings of other studies,[18,20,21] although a recent study found that females with higher incomes were more satisfied with primary health services.[30]

Several studies have reported that a comfortable physical environment was a factor in encouraging utilization of the services and increasing satisfaction. The main concern of the consumers in this study was the lack of space in the waiting area. The areas around the holy shrines are very limited, which gives concern to the health services and all other services. It is hoped that there would be more space made available in future.

The subjects were also concerned about many aspects of pharmacy and the services of pharmacist. There was poor satisfaction with pharmacy services because pharmacists had failed to give sufficient information on the side effects of drugs and precautions that need to be taken. Pharmacists should play an active role in providing information on drug interactions and side effects and should be ready to answer patient’s queries. Many studies have shown that pharmacy services in ambulatory settings are a cause of concern to many consumers.[20,21] Results of this study appear to support findings of some studies which showed that the less-educated consumers were more satisfied than the more educated.[19,31–34] Many other studies did not find significant consistent association between education and satisfaction.[18,21,29]

Consumers who work at semiskilled or unskilled jobs were significantly more satisfied in the present study, while a previous study in Riyadh found that the highest satisfaction score was among teachers (96%).[29]

In a previous study in Riyadh city, married consumers were more satisfied than those who were single.[29] Our findings in only a few services appeared to agree with this. Widowed subjects tended to have higher satisfaction with most services in our study, but the differences were not significant. Many studies have not shown any significant relationship between marital status and satisfaction.[18–22]

The results of this study, in general, revealed no significant consistent pattern of association of satisfaction with the variables studied, except for occupation and education. Evidently, patients’ socio-demographic variables have been studied in several communities but their relation to opinions and attitudes, and their association with satisfaction have been rather inconsistent and at times contradictory.[30–35] Several factors are associated with the positive attitudes and satisfaction of patients with the health services. These include manpower characteristics, resources and service organization, distance travelled, travel and waiting times, working hours, and the attitudes of the patient towards life itself. Also, methodology of the study and whether self-administered or interviews were used to collect data, the types, number and sequence of questions asked, the timing and setting may all have affected the level of satisfaction. Although studies may be costly, information gathered on consumers’ opinions about the services provided, the methods used, can lead to many changes that may be beneficial to the consumers, the health team, and the entire health system.

## CONCLUSION

There was acceptable satisfaction with ambulatory health services for the Hajj. Problems with physicians and services in the waiting area need to be addressed in order to raise the satisfaction level with ambulatory health services during future Hajj seasons. We recommend a larger scale more comprehensive study covering all levels of health services.

### Study limitation

Because of the special circumstances of the Hajj, the questionnaire did not include all parameters affecting satisfaction. The sample size and duration of study were also affected by the special status and duration of Hajj season. The study design was cross sectional with its limitations. Patients’ feedbacks were subjective.
